# Identification and functional characterization of circRNA-0008717 as an oncogene in osteosarcoma through sponging miR-203

**DOI:** 10.18632/oncotarget.23466

**Published:** 2017-12-20

**Authors:** Xiang Zhou, Dimple Natino, Zili Qin, Dong Wang, Zhen Tian, Xuan Cai, Bo Wang, Xijing He

**Affiliations:** ^1^ Department of Orthopaedics, Second Affiliated Hospital of Xi’an Jiaotong University, Xi'an, China; ^2^ Department of Biomedical Sciences, Florida State University College of Medicine, Tallahassee, FL, USA; ^3^ Department of Otolaryngology, Otorhinolaryngology Hospital, The First Affiliated Hospital of Sun Yat-sen University, Guangzhou, China; ^4^ Department of Infectious Diseases, First Affiliated Hospital of Xi’an Jiaotong University, Xi'an, China; ^5^ Department of Pharmacology, School of Basic Medical Science, Xi’an Jiaotong University Health Science Center, Xi'an, China

**Keywords:** osteosarcoma, circRNA-0008717, miR-203, Bmi-1

## Abstract

Circular RNA (circRNA) is a key regulator in the development and progression of human cancers, however its role in osteosarcoma tumorigenesis is not well understood. The present study aims to investigate the expression profiles and potential modulation of circRNA on osteosarcoma carcinogenesis. Human circRNA microarray was performed to screen for abnormally expressed circRNA in osteosarcoma tissue and circRNA-0008717 was identified as one circRNA significantly upregulated in osteosarcoma tissue. Osteosarcoma patients with high circRNA-0008717 expression had shortened survival. Gain and loss functional assays suggested that knockdown of circRNA-0008717 suppressed cell proliferation, migration and invasion, but promoted cell apoptosis. By using biotin-labeled circRNA-0008717 probe to perform RNA precipitation in osteosarcoma cells, we identified miR-203 as the circ0008717-associated microRNA. Subsequently, Bmi-1 was identified as the functional target of miR-203. In addition, overexpression of circRNA-0008717 in osteosarcoma could elevate Bmi-1 expression, resulting in the promotion of osteosarcoma cell proliferation and invasion. Furthermore, the tumor promoting effect of circRNA-0008717 was abolished by miR-203 mimics or Bmi-1 silencing vector. In conclusion, circRNA-0008717 plays an oncogenic role in osteosarcoma and may serve as a promising prognostic biomarker for osteosarcoma patients. Therefore, silence of circRNA-0008717 could be a future direction to develop a novel treatment strategy.

## INTRODUCTION

Osteosarcoma is the most common primary bone malignancy in children and young adults, and accounts for approximately 60% of malignant bone tumors in the first two decades of life [1]. Currently, pulmonary metastasis is the most common cause for cancer-related death [2]. Despite advancement strategies such as surgery, adjuvant chemotherapy, and radiotherapy, the prognosis of osteosarcoma still remains poor, and the survival of osteosarcoma patients reached a plateau [3–5]. Genetic changes as well as dysfunction of oncogenes or tumor suppressors have been demonstrated to be tightly associated with the development and progression [6, 7]. Hence, identification of new molecules involved in tumor progression is of crucial importance to reduce the morbidity and mortality of this devastating disease.

Circular RNAs (circRNAs) are a class of non-coding RNAs characterized by covalently closed continuous loops with neither 5′ to 3′ polarity nor a polyadenylated tail [8]. They have been found to play an important role in the regulation of multiple diseases, including cancer [9, 10]. Recently, certain kinds of circRNAs have been found to be significantly deregulated in gastric cancer, esophageal squamous cancer, and osteosarcoma, and these deregulated circRNAs is suggested to participate in cancer development [11–13].

Accumulating evidences demonstrate that circRNAs function as microRNA (miRNA) sponges, RNA-binding protein (RBP) sequestering agents as well as transcription regulators to regulate gene expressions [14–17]. For example, circRNA CDR1as, also known as ciRS-7, has been identified as a miR-7 sponge, leading to inhibition of miR-7 activity and activating the targeted gene expression [18]. Compared with linear RNA, circRNA is composed of a ring structure that is hard to break down. Thus, circRNA remains stable and serves as a convenient tool for cancer monitoring. These observations indicate that circRNAs may be a new kind of potential biomarkers and therapeutic targets for cancer; however, elucidating the deregulated circRNAs and identifying their functions are still an ongoing process in cancer investigation.

MicroRNAs (miRNAs) are a novel class of endogenous, small, non-coding RNAs that exert their posttranscriptional regulatory effects by targeting 3′ untranslated region (3′-UTR) of corresponding mRNAs [19]. By targeting multiple transcripts, miRNAs regulate a wide range of biological processes, including apoptosis, differentiation and cell proliferation. Deregulation of miRNA expression by the sponge function of circRNAs may be critical for the development of cancers [20].

In the present study, we utilized circRNA microarray analysis to screen expression profiles of circRNAs in osteosarcoma specimens, and identified that circRNA-0008717 is significantly upregulated and predicts poor prognosis of osteosarcoma patients. We found that circRNA-0008717 plays a oncogenic role in osteosarcoma via the sponge of miR-203 to promote thymidylate synthase expression, and consequently suppress osteosarcoma progression. Hence, circRNA-0008717 may serve as a biomarker for prognosis predication and as a potential therapeutic target for osteosarcoma patients.

## RESULTS

### CircRNAs expression profiles analysis

**Figure 1:**
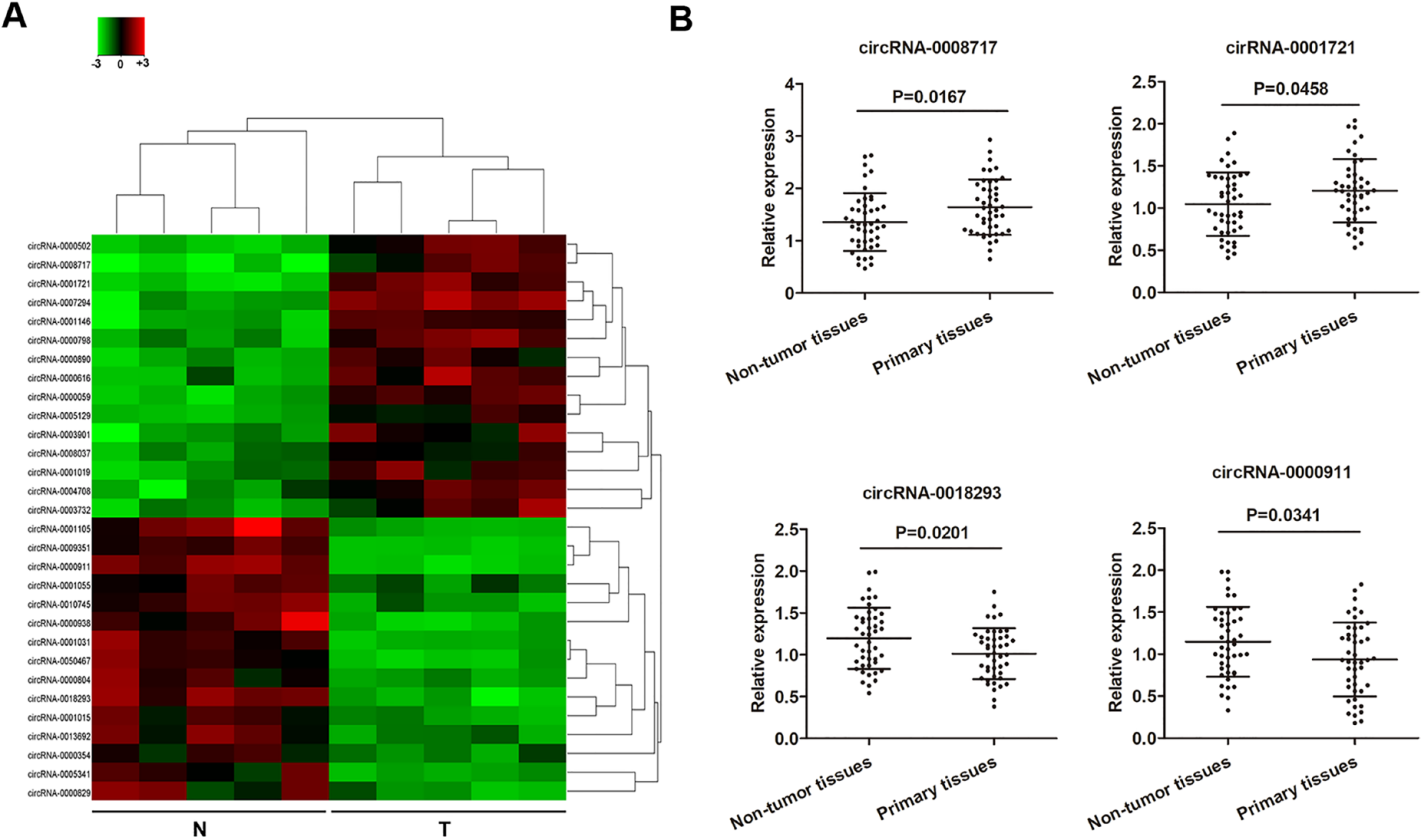
Deregulated circRNAs in osteosarcoma tumor tissues (**A**) The heat map showed the top fifteen most increased and decreased circRNAs in osteosarcoma tissues as compared to that in the matched non-tumor tissues analyzed by circRNAs Arraystar Chip. (**B**) Relative expression of the four indicated circRNAs from 45 osteosarcoma tumor tissues and adjacent non-tumor tissues listed in (A) measured by RT-qPCR. N, non-tumor tissues; T, tumor tissues.

To identify specific circRNAs that are differentially expressed between osteosarcoma patients and normal subjects, five tissues samples and matched non-tumor tissue samples pooled from osteosarcoma patients were subjected for circRNA microarray assay analysis. A total of 677 circRNAs that were differently expressed more than 2-fold change between osteosarcoma tissues and normal tissues were identified. Subsequently, we narrowed the scope of the study to the 30 most aberrant expressed circRNAs, including 15 upregulated circRNAs and 15 downregulated circRNAs (shown in the heat map, Figure 1A). We then detected the expression of these 30 mostly changed circRNAs in ostesoarcoma and matched non-tumor tissue samples from 45 patients to confirm their expression. As shown in Figure 1B, four circRNAs were found to be significantly deregulated between osteosarcoma and paired noncancerous tissues (*P <* 0.05), including two upregulated cirRNAs, cirRNA-0008717 and cirRNA-0001721, and two downregulated circRNAs, circRNA-0018293 and circRNA-0000911.

### CircRNA-008717 was upregulated in osteosarcoma patients and correlated with poor survival

**Figure 2:**
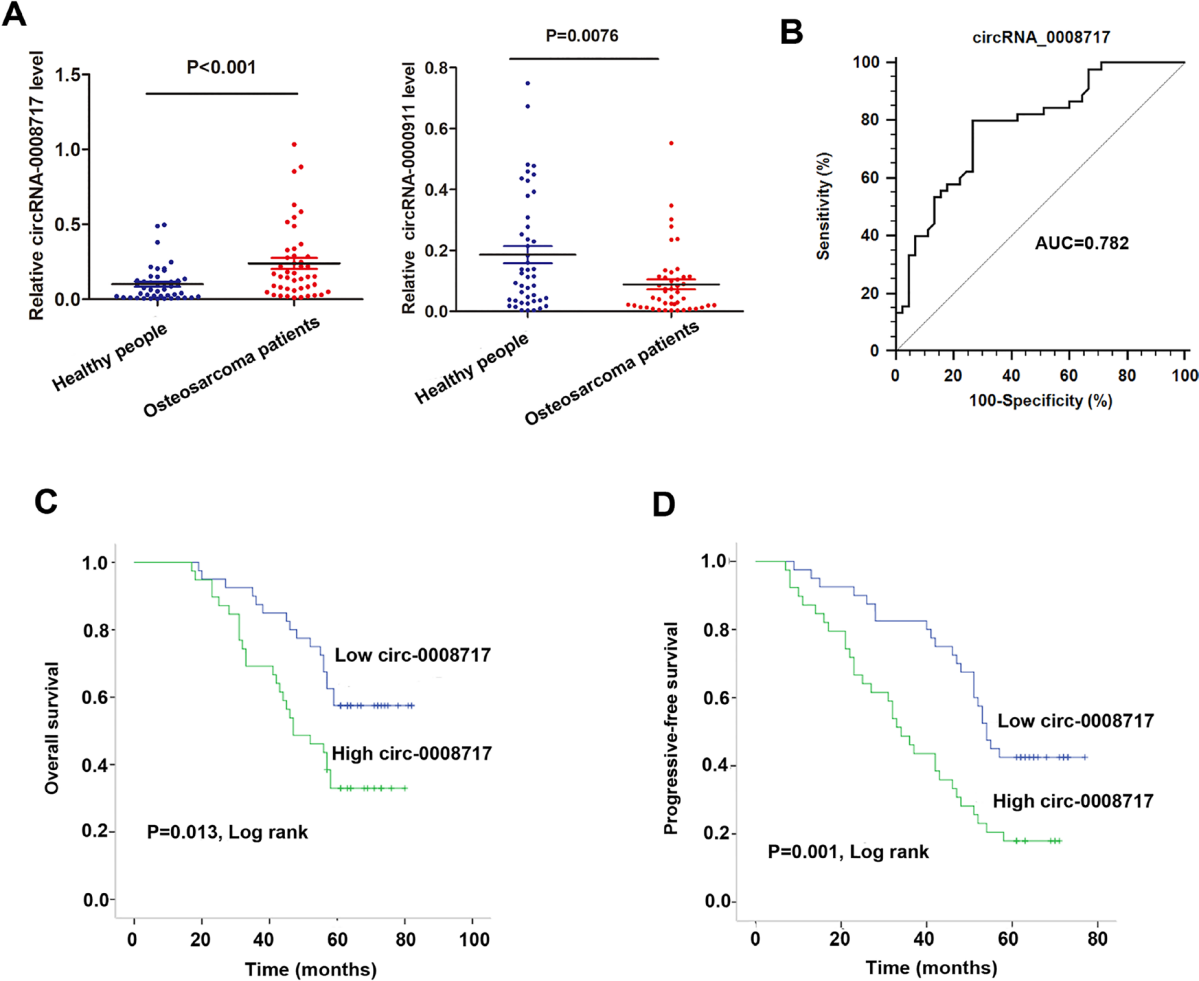
CircRNA-0008717 was upregulated in osteosarcoma patients and correlated with poor survival (**A**) The expression level of circRNA-0008717 and circRNA-0000911 were detected in serum samples from 45 osteososarcoma patients and 45 healthy individuals. (**B**) ROC curve analysis was performed to investigate the diagnostic value of circRNA-0008717 for osteosarcoma patients. (**C**–**D**) Kaplan-Meier curves for overall survival (C) and progressive-free survival (D) were drawn according to circRNA-0008717 expression in 45 primary osteosarcoma tissues and were analyzed by using log-rank test.

The expression of the four circRNAs were then validated in another independent cohort of serum samples from 45 osteosarcoma patients and 45 healthy individuals. RT-qPCR assay showed that only cirRNA-0008717 and circRNA-0000911 maintained their statistical significance between the two groups (Figure 2A). However, our preliminary results showed that circRNA-0000911 was normally expressed in osteosarcoma cells and ectopic expression of circRNA-0000911 had no significant effect on osteosarcoma cell viability (data not shown), and only silencing of circ-0008717 had a significant effect to suppress cell growth. This inspired us to focus on the biological significance of circRNA-0008717 in osteosarcoma progression.

Receiver operation curve (ROC) analysis was performed by using the 45 paired tissues samples to investigate the diagnostic value of circRNA-0008717. As shown in Figure 2B, the area under the ROC curve (AUC) of circRNA-0008717 was 0.782 (95% CI: 0.682–0.862) and the diagnostic sensitivity and specificity reached 80.00% and 73.33%, respectively. On the basis of the cut-off established by the ROC analysis (0.97), we divided the 45 patients into a high-circRNA0008717 expressing group and a low expressing group. Kaplan-Meier survival curve showed that patients with high expression of circRNA-0008717 was significantly correlated with poor overall survival (OS) and progressive-free survival (PFS) (Figure 2C and 2D). Furthermore, we performed Cox regression univariate/mutivariate analysis to identify whether circRNA-0008717 was an independent indicator for OS of osteosarcoma patients. As shown in Table 1, circRNA-0008717 expression level and pulmonary metastasis maintained their significance as independent prognostic factors for OS of osteosarcoma patients.

**Table 1:** Univariate and multivariate Cox proportional hazards regression model analysis for overall survival in osteosarcoma patients

Characteristics	Univariate analysis			Multivariate analysis		
	HR	95% CI	*P* value	HR	95% CI	*P* value
Gender	0.998	0.645–2.854	0.632			
Age	1.182	0.636–3.438	0.284			
Tumor size	1.703	0.654–3.660	0.378			
Differentiation	1.824	0.695–3.436	0.209			
TNM stage	2.008	1.134–3.741	0.044	2.303	1.178–4.274	0.060
Pulmonary metastasis	3.417	1.564-7.137	0.009	3.597	1.499–7.902	0.007
circRNA-0008717 expression	3.452	1.447–4.860	0.013	3.505	1.287–5.221	0.011

### CircRNA-0008717 plays an oncogenic role in osteosarcoma cells

**Figure 3:**
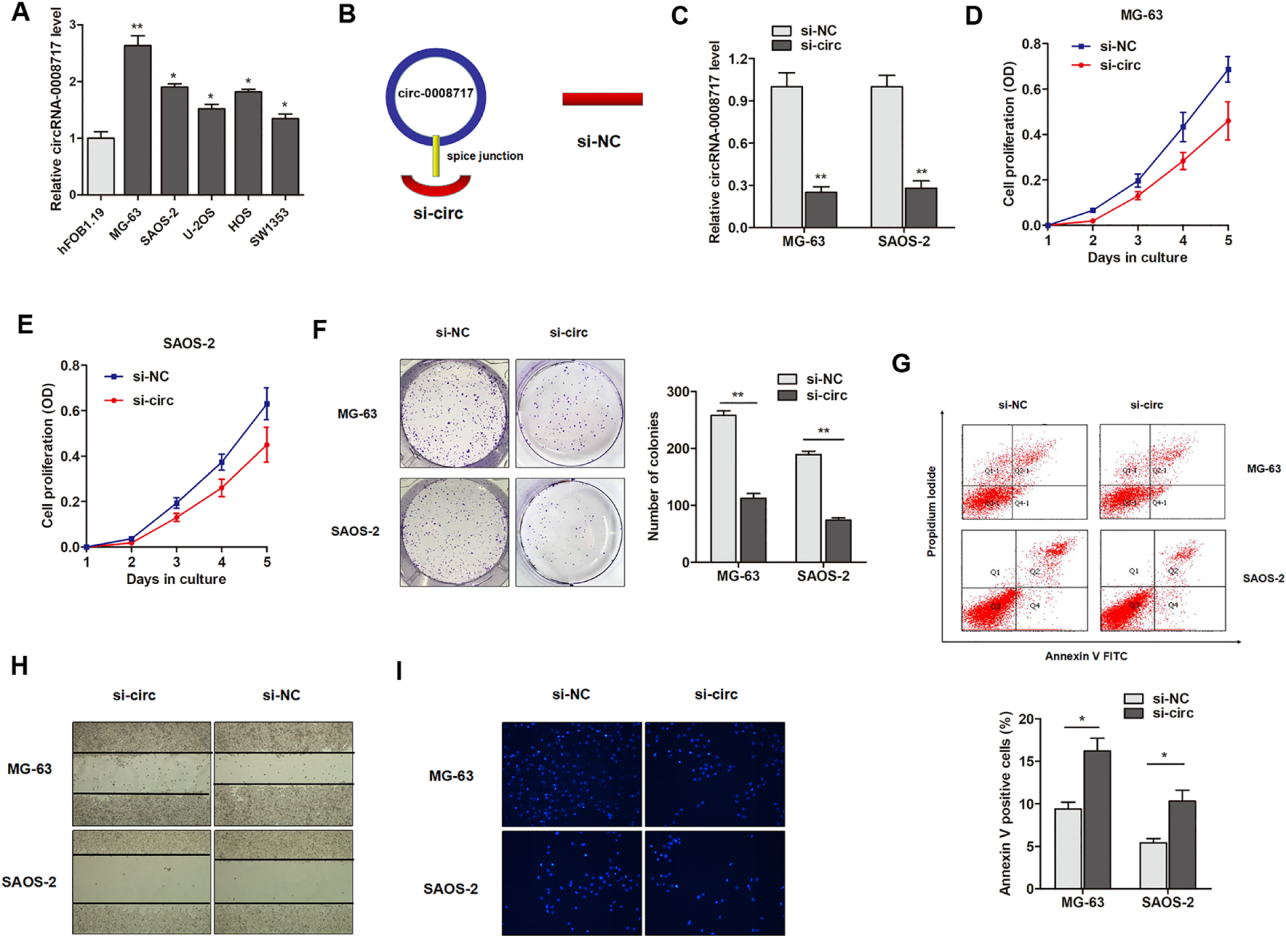
CircRNA-0008717 plays an oncogenic role in osteosarcoma cells (**A**) The expression level of circRNA-0008717 in five osteosarcoma cell lines and one normal osteoblastic cell line hFOB. (**B**) The sketch of structures of si-circ, si-NC vector are shown. (**C**) CircRNA-0008717 was silenced by transfection of specific silencing vectors. (**D–E**) MTT assay showed that cell proliferation was dramatically inhibited by knockdown of circRNA-0008717 in MG-63 (D) and SAOS-2 (E) cells. (**F**) Knockdown of circRNA-0008717 significantly suppressed the colony formation capacity of both osteosarcoma cells. (**G**) FACS apoptosis assay indicated that circRNA-0008717 silencing promoted the proportion of apoptotic cells in both osteosarcoma cell lines. (**H**–**I**) Wound-healing (H) and Matrigel invasion (I) assay suggested that knockdown of circRNA-0008717 suppressed the migration and invasion abilities of osteosarcoma cells.

The role of circRNA-0008717 in osteosarcoma progression was then investigated. As shown in Figure 3A, the expression of circRNA-0008717 was upregulated in most osteosarcoma cell lines when compared with the osteoblastic cell line hFOB. MG-63 and SAOS-2 cell lines were chosen for further investigations, because these two cell lines showed relatively higher expression level of circRNA-0008717 among the osteosarcoma cell lines. Then, the short interfering RNA (siRNA) which covers back-splicing region of circRNA-0008717 was constructed (Figure 3B). RT-qPCR assay showed that si-circ vector but not si-NC significantly downregulated the endogenous levels of circRNA-0008717 in MG-63 and SAOS-2 cells (Figure 3C). Subsequently, the effects of circRNA-0008717 on osteosarcoma cell proliferation, apoptosis, invasion and migration were examined. MTT assay showed that knockdown of circRNA-0008717 significantly suppressed cell proliferation when compared with control vector in both cell lines (Figure 3D and 3E). Similarly, decreased expression of circRNA-0008717 inhibited the colony formation ability, and promoted the proportion of apoptotic cells (Figure 3F and 3G). In addition, si-circ significantly inhibited the wound healing ability of osteosarcoma cells (Figure 3H). Matrigel invasion assay showed that silence of circRNA-0008717 noticeably suppressed invasive capabilities of both cells (Figure 3I).

### CircRNA-0008717 directly binds to miR-203 in osteosarcoma cells

**Figure 4:**
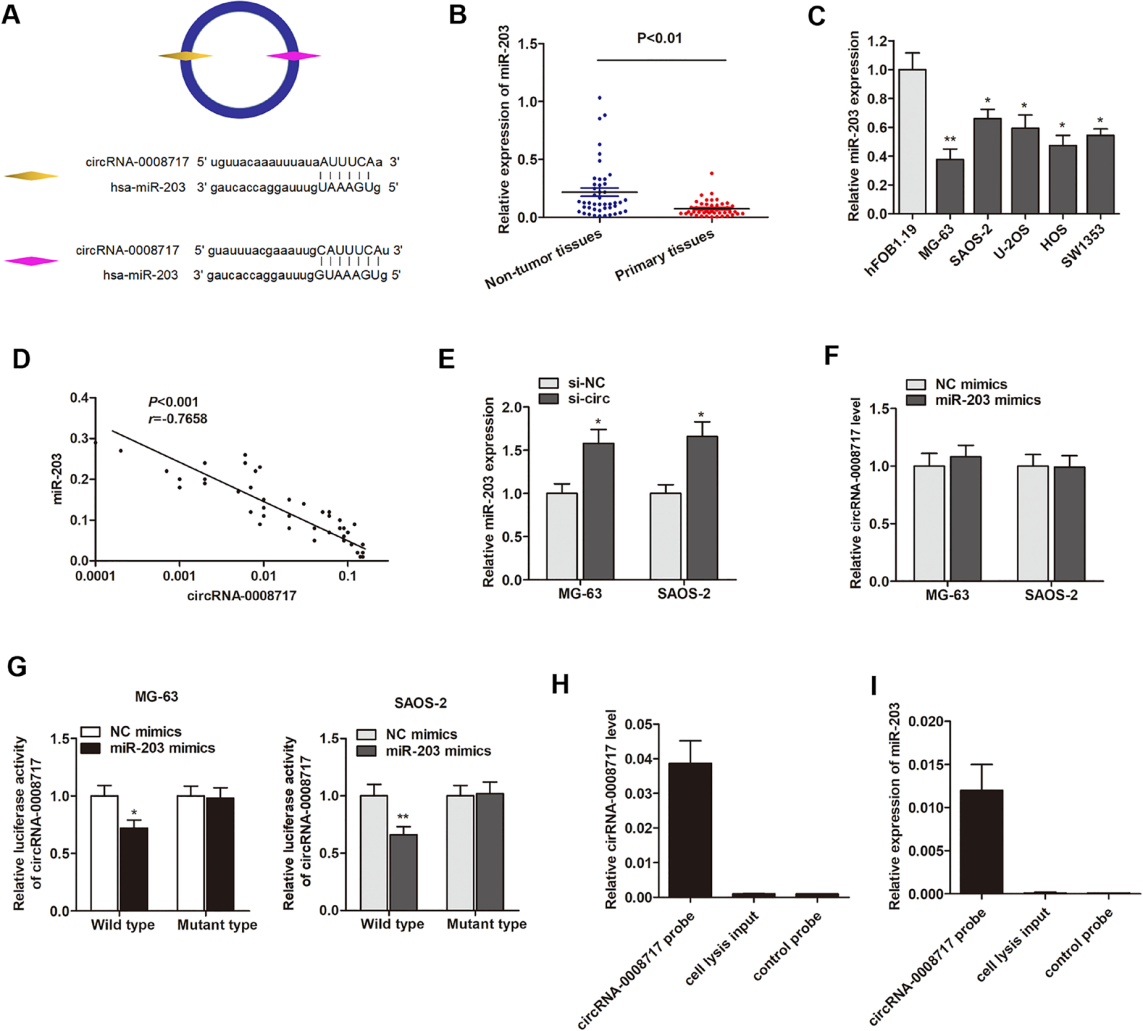
CircRNA-0008717 directly binds to miR-203 in osteosarcoma cells (**A**) The putative sequences of miR-203 and circRNA-0008717 with two binding sites. (**B**) MiR-203 was downregulated in 45 primary osteosarcoma tissues in contrast to paired non-tumor tissues. (**C**) MiR-203 was downregulated in osteosarcoma cell lines. (**D**) Spearman correlation testing indicated circRNA-0008717 was negatively correlated with miR-203 expression level in 45 primary osteosarcoma tissues. (**E**) MiR-203 was upregulated in osteosarcoma cells that transfected by si-circ. (**F**) MiR-203 showed no influence on the expression level of circRNA-0008717. (**G**) MiR-203 significantly inhibited luciferase activity of wild type reporter for cirRNA-0008717, however, miR-203 did not inhibit the luciferase activity of reporter vector containing the mutant binding sites of cirRNA-0008717. (**H**) CircRNA-0008717 in MG-63 cell lysis was pulled down and enriched with circRNA-0008717 specific probe and then detected by RT-qPCR. (**I**) MiR-203 was pulled down and enriched with circRNA-0008717 specific probe and then detected by RT-qPCR.

Because circRNAs function mainly as miRNA sponges to bind to functional miRNAs and then regulate gene expression, we next examined the potential miRNAs associated with circRNA-0008717. According to miRBase prediction, circRNA-0008717 possessed two complementary sequences to miR-203 seed region (Figure 4A). RT-qPCR showed that miR-203 was significantly downregulated in osteosarcoma tissues and cell lines (Figure 4B and 4C). Spearman correlation test suggested that circRNA-0008717 was negatively correlated with miR-203 expression (Figure 4D). Moreover, knockdown of circRNA-0008717 significantly promoted miR-203 expression (Figure 4E), however, miR-203 failed to influence cirRNA-0008717 level (Figure 4F). Subsequently, luciferase reporter assay was performed to verify that miR-203 directly interacted with circRNA-0008717. As shown in Figure 4G, miR-203 significantly inhibited luciferase activity of wild type reporter for cirRNA-0008717, however, miR-203 did not inhibit the luciferase activity of reporter vector containing the mutant binding sites of cirRNA-0008717. Finally, we used cirRNA-0008717 specific probe to perform RNA precipitation (RIP). The cirRNA-0008717-associated RNAs were purified by cirRNA-0008717 specific probe, and then screened the miRNAs that were pulled down by cirRNA-0008717 with RT-qPCR assay. As expected, we found a specific enrichment of cirRNA-0008717 and miR-203 as compared to the controls (Figure 4H and 4I), indicating that cirRNA-0008717 specifically interacts with miR-203 in osteosarcoma cells.

### miR-203 targets BMI-1 and suppresses osteosarcoma cell proliferation and invasion

**Figure 5:**
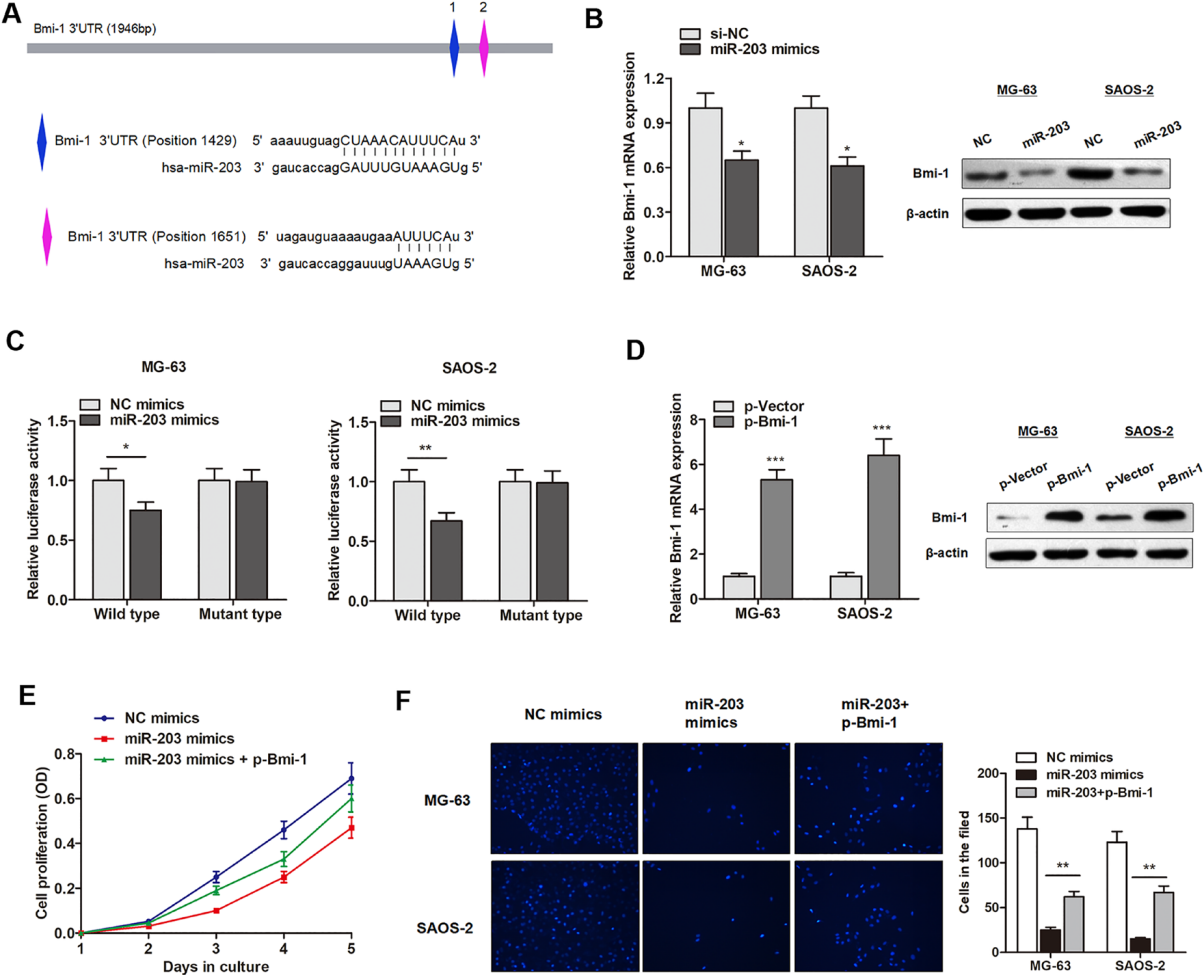
miR-203 targets BMI-1 and suppresses osteosarcoma cell proliferation and invasion (**A**) The putative sequences of miR-203 and Bmi-1 with two binding sites. (**B**) Bmi-1 mRNA and protein levels were downregulated by miR-203. (**C**) MiR-203 significantly inhibited luciferase activity of wild type reporter for Bmi-1, however, miR-203 did not inhibit the luciferase activity of reporter vector containing the mutant binding sites of Bmi-1 in both cell lines. (**D**) Bmi-1 overexpressing vector significantly promoted Bmi-1 expression level in both transcript and protein level. (**E**–**F**) MiR-203 mimics suppressed proliferation rate (E) and invasive capacity (F) of osteosarcoma cells, however, this effect was dramatically reversed by cotransfection with Bmi-1.

According to miRBase prediction, miR-203 could target Bmi-1 3′UTR with a high score (Figure 5A). RT-qPCR and western blot assay showed that miR-203 mimics inhibited Bmi-1 expression at both transcript and protein level, respectively (Figure 5B). To further identify whether Bmi-1 in osteosarcoma cells responded to miR-203 through direct interactions with its 3′-UTR, we cloned the wild type 3′-UTR of the putative miR-203 target or mutant sequences into reporter plasmid downstream of the luciferase gene. The Dual-Luciferase reporter assay showed that the miR-203 mimics attenuated the fluorescence driven by the wild type 3′-UTR by more than 1.5-fold compared with the negative control, whereas the mutant 3′-UTR was not affected by miR-203 (Figure 5C).

Subsequently, we evaluated the effect of miR-203 on osteosarcoma cell proliferation and invasion. Bmi-1 was upregulated in osteosarcoma cells by Bmi-1 overexpression vector (Figure 5D). As shown in Figure 5E–5F, miR-203 mimics suppressed proliferation rate and invasive capacity of MG-63 cells, however, this effect was dramatically reversed by cotransfection with Bmi-1. This suggests that miR-203 suppresses osteosarcoma progression through targeting Bmi-1.

### CircRNA-0008717 promotes osteosarcoma progression by sponge activity of miR-203 and upregulation of Bmi-1

**Figure 6:**
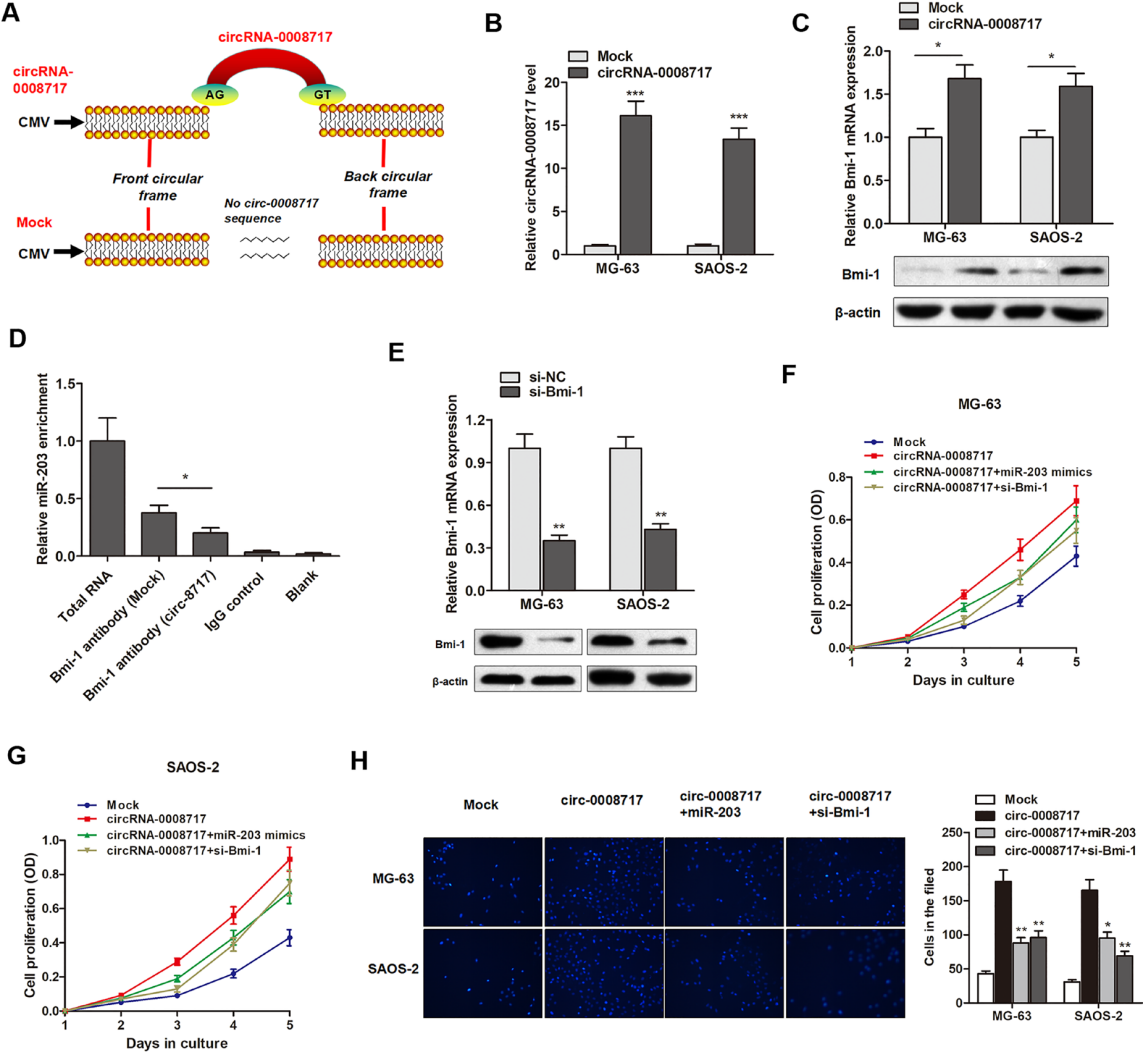
CircRNA-0008717 promotes osteosarcoma progression by sponge activity of miR-203 and upregulation of Bmi-1 (**A**) The sketch of structures of circRNA-0008717 and mock vector are shown. (**B**) CircRNA-0008717 are upregulated by transfection of circRNA vector. (**C**) Both Bmi-1 transcript and protein levels were upregulated by overexpression of circRNA-0008717. (**D**) RIP experiments were performed using the Bmi-1 antibody to immunoprecipitate RNA and a primer to detect miR-203, and a significantly decreased enrichment of miR-203 was identified in cells transfected with circRNA-0008717 vector. (**E**) Bmi-1 was silenced by specific siRNAs at both transcript and protein levels. (**F**–**G**) CircRNA-0008717 significantly promoted cell proliferation rate, however, this effect was significantly abrogated by cotransfection with miR-203 mimics or si-Bmi-1 vector. (**H**) The enhanced cell invasive capacity of both cell lines induced by circRNA-0008717 was abolished by cotransfection with miR-203 mimics or si-Bmi-1 vector.

After having validated that Bmi-1 was a direct target of miR-203, we sought to determine whether circRNA-0008717 promoted Bmi-1 expression through sponging miR-203. We first constructed the circular transcript expression vector. The full-length cDNA of circRNA-0008717 from MG-63 cells was amplified and cloned into the specific vector (Figure 6A). RT-qPCR showed that circRNA-0008717 vector significantly elevated the level of circRNA-0008717 in both cells (Figure 6B). Gain and loss functional assay indicated that enhanced circRNA-0008717 promoted Bmi-1 expression at both transcript and protein levels (Figure 6C). Furthermore, RIP showed that the enrichment of Bmi-1 and miR-203 was significantly decreased in MG-63 cells transfected with circRNA-0008717 vector (Figure 6D).

Take a step further, we sought to determine that circRNA-0008717 regulates cell proliferation and invasion through sponging miR-203 and subsequently elevating Bmi-1. Bmi-1 silencing vector was then constructed (Figure 6E). As shown in Figure 6F and 6G, circRNA-0008717 significantly promoted cell proliferation rate, however, this effect was significantly abrogated by cotransfection with miR-203 mimics or si-Bmi-1 vector. Similarly, the enhanced cell invasive capacity of both cell lines induced by circRNA-0008717 was abolished by cotransfection with miR-203 mimics or si-Bmi-1 vector (Figure 6H).

## DISCUSSION

Despite the rapid development of early diagnosis and treatment in osteosarcoma, invariably, nearly all osteosarcoma patients finally become metastatic and chemoresistant [21]. It is widely accepted that searching new therapeutic targets and better understanding the pathway related to cancer initiation and progression is essential for improving the prognosis of osteosarcoma patients. In this study, we focused on a novel gene regulator, circRNA, and validated the upregulation of circRNA-0008717 in osteosarcoma specimens. Furthermore, we also uncovered that the increased expression of circRNA-0008717 was a potential diagnostic and prognostic factor for osteosarcoma patients. Mechanistic analysis indicated that circRNA-0008717 suppressed the capacity of proliferation, migration and invasion of osteosarcoma cells through specifically sponging miR-203 and releasing Bmi-1.

The concept of “circular RNA” was first proposed in 1976, Sanger and colleagues found that viroids are single-stranded covalently closed circRNA molecules pathogenic to certain higher plants [22]. CircRNAs are an enigmatic class of RNA with unknown function in mammal cells, and often show tissue/developmental-stage-specific expression [23, 24]. They have two significant properties: highly conserved and remarkably stable with a half-life more than 48 hours [25]. Harboring these properties, circRNAs may be a promising tumor marker in precision medicine. Previously, increasing researches reveal the strong association of circRNAs with the development of several diseases, such as Alzheimer's disease, type 2 diabetes mellitus, and different types of cancer, which make circRNA with the feasible potential of serving as the molecular diagnostic biomarker [26]. However, the specific role of circRNA in osteosarcoma is still largely unknown. In the present study, a circRNA microarray was performed to detect the circRNA expression profiles of osteosarcoma tissues and paired nontumor tissues to explore the relationship between the circRNA expression and osteosarcoma and to provide a preliminary theoretical basis for search of biomarkers for the early diagnosis and malignant progression of osteosarcoma. We identified that circRNA-0008717 was upregulated in osteosarcoma. ROC curve analysis and subsequent Kaplan-Meier analysis revealed that circRNA-0008717 may be a promising diagnostic and prognostic biomarker for osteosarcoma patients.

After having verified the clinical value of circRNA-0008717, we then investigate the underlying regulatory mechanism that may account for the clinical findings. CircRNA-0008717 is located at chr1:229665945–229678118 with 724 length in gene symbol ABCB10, thus, it was also labeled circ-ABCB10. One study by Liang et al have demonstrated that this circRNA can promote breast cancer proliferation and progression through the sponge of miR-1271 function [27]. However, the functional role of circRNA-0008717 was not reported in other type of cancers, including osteosarcoma. In this study, we demonstrated that knockdown of circRNA-0008717 suppressed cell proliferation, migration and invasion, and promoted cell apoptosis; while enhanced expression of circRNA-0008717 promoted cell proliferation and invasion, indicating that this circRNA plays a oncogenic role in osteosarcoma.

MiRNAs, an abundant class of small noncoding RNAs (~22 nt), posttranscriptionally modulate the translation of target mRNAs via corresponding miRNA response elements (MRE) [28]. Computational searches for miRNA target sites in circRNAs identified a portion of circRNA molecules that contain MREs, which might act as miRNA sponge, reducing miRNA binding to its target genes, thereby releasing the expression of the miRNA targets indirectly [29–32]. Since the first report of circRNA functioning as a miRNA sponge, the potential of circRNAs in regulating cancer-related genes through fine-tuning miRNAs has recently been recognized. For example, the first characterized circRNA to support this model is ciRS-7, which contains more than sixty miR-7-binding sites, thereby acting as an endogenous miRNA sponge to adsorb and subsequently quench normal miR-7 functions [33, 34]. More recently, more and more circRNAs were recognized as miRNA sponges in different cancers, such as colorectal cancer, gastric cancer, breast cancer and hepatocellular carcinoma [35–37]. By performing luciferase reporter assay, RIP assay and other gain and loss-functional assays, we demonstrate that circRNA-0008717 play an oncogenic role in osteosarcoma through the sponge activity of miR-203.

MiR-203, located at the chromosome 14q32.33, is one of the most frequently mentioned miRNAs. Previous studies have showed that miR-203 exhibited aberrant expression in multiple malignancies compared with their normal tissues [38]. Although the role of miR-203 in malignancies are conflicting, miR-203 acts as a tumor suppressor in tumor progression of osteosarcoma [39]. We also validated that miR-203 was silenced in osteosarcoma and the downregulated miR-203 suppressed cell proliferation and invasion. By performing bioinformatics analysis and subsequent functional validation, we identified Bmi-1 as the direct target of miR-203. As a key member of the PcG complex, Bmi-1 was originally identified as an oncogene cooperating with c-myc in murine lymphomas model. It contains a conserved N-terminal RING finger domain and a central HTHTHT motif, which is necessary for telomerase activity induction and cellular immortalization [40]. Recently, Bmi-1 overexpression has been found in a variety of malignant tumors [41], and silence of Bmi-1 expression can cause apoptosis and senescence of tumor cells [42], suggesting an important role in tumor cell growth and survival. Our study also suggests that Bmi-1 is necessary for the anti-oncogenic role of miR-203, which is consistent with the previous studies.

Finally, our gain and loss-functional assays verified that circRNA-0008717 promotes osteosarcoma progression by sponge activity of miR-203 and upregulation of Bmi-1 expression. To the best of our knowledge, this is the first study to systematically identify the expression and functional role of circRNA-0008717 in osteosarcoma. We reported that circRNA-0008717 is upregulated and predicts poor survival in osteosarcoma patients. Moreover, circRNA-0008717 acts as a tumor oncogene through sponging miR-203 and thereby promoting the function of Bmi-1. Therefore, circRNA-0008717 may serve as a promising predictive biomarker and therapeutic target for osteosarcoma patients.

## MATERIALS AND METHODS

### Clinical samples and preparation

Forty-five cancer tissues and paired adjacent noncancerous tissues from primary osteosarcoma patients were collected at Second Affiliated Hospital of Xi’an Jiaotong University between June 2012 and July 2014. Another independent cohort of serum samples from 45 osteosarcoma patients and 45 healthy controls were also enrolled between June 2012 and July 2014, for the validation of date obtained from tissue samples. All the patients were pathologically confirmed and they were classified according to the tumor-node-metastasis (TNM) classification. OS was updated on 1 February 2012 and was defined as the time from inclusion to death for any reason. RFS was defined as the time from inclusion to recurrence or metastasis progression. Tissues specimens were obtained during operation and immediately frozen at –80°C until RNA extraction. Venous blood was collected and centrifuged at 4000 rpm for 10 min, within 2 h. The supernatant fluids were then collected and further centrifuged at 12000 rpm for 15 min to completely remove the cell debris. The whole process was strictly controlled to avoid hemolysis, and the supernatant serum was stored at –80°C, until further used. Written informed consent was obtained from all patients according to the guidelines approved by the Ethics Committee of the Second Affiliated Hospital of Xi’an Jiaotong University.

### Cell culture

Human osteosarcoma cell lines MG-63, SAOS-2, U-2OS, HOS, SW1353 and one osteoblastic cell line (hFOB1.19) were obtained from the Type Culture Collection of the Chinese Academy of Sciences (Shanghai, China). All osteosarcoma cell lines were maintained in Dulbecco's Modified Eagle's Medium (DMEM) medium (Invitrogen, Carlsbad, CA, USA) containing 10% fetal bovine serum (FBS) (Sigma-Aldrich, St. Louis, MO, USA), 100 U/ml penicillin and 100 g/ml streptomycin (Life Technologies, Grand Island, NY, USA) at 37°C in 5% CO_2_ and 95% air. Osteoblastic hFOB cells were grown in DMEM/F12 1:1 medium with 10% FBS, 2.5 mM L-glutamine and 0.3 mg/ml G418 at 37°C in 5% CO2 and 95% air. The cell lines passed the DNA profiling test (Short Tandem Repeat, STR).

### Expression profile analysis of circRNAs

The circRNAs chip (Arraystar Human circRNAs chip, ArrayStar) containing 5396 probes specific for human circular RNAs splicing sites was used. After hybridization and washing with samples, five pairs of osteosarcoma samples (tumor tissues and matched non-tumor tissues) were analyzed on the circRNAs chips. Exogenous RNAs developed by ERCC (External RNA Controls Consortium) were used as controls. The GEO Accession number is GSE97332.

### RNA oligoribonucleotides and cell transfection

The miR-203 mimics, and small interfering RNAs (siRNAs) that specifically target Bmi-1 (si-Bmi-1) was synthesized by GenePharma (Shanghai, China). The circular transcript expression vector possesses two elements termed as the front circular and the back circular frame which were specially designed containing inverted repeat sequences flank. The full-length cDNA of circRNA-0008717 was amplified in MG-63 cells, and was cloned into the specific vector between two frames; while, the mock plasmid without the circRNA-0008717 cDNA was served as control. To knockdown circRNA-0008717, three siRNA against circRNA-0008717 and siRNA-NC were synthesized, and the efficiency was examined using RT-qPCR in MG-63 and SAOS-2 cells. The most effective siRNA (siRNA-2) was determined for synthesizing shRNA. siRNA-circ: 5′-TAGAAGACCATGGGGGATGTCAAGAGCATCCCCCATGGTCTTCTATTTTTT-3′.siRNA-NC: 5′-TTCTCCGAACGTGTCACGTTCAAGAGACGTGACACGTTCGGAGAATTTTTT-3′. The cDNA sequence of circRNA-0008717 was synthesized by the Ribo Bio Company (Guangzhou, China) and then cloned into the lentiviral expression vector pLVXIRES-neo, (Clontech Laboratories Inc., San Francisco, CA, USA). Lentiviral production and transduction were conducted by following previously published procedures [43]. Osteosarcoma cells were maintained in a 6-well plate in DMEM supplemented with 10% FBS and cultured until 50–70% confluent. RNA oligoribonucleotides were mixed with Lipofectamine 3000 (Invitrogen, Carlsbad, CA, USA) in reduced serum medium (Opti-MEM, Gibco, USA) according to the manufacturer's instructions and final concentration of RNA oligoribonucleotides was 100 nM. Knockdown or overexpression effect was examined by RT-qPCR using RNA extracted 48 hours after transfection.

### Quantitative real-time PCR (RT-qPCR)

Total RNA was isolated from primary osteosarcoma specimens or cell lines using TRIzol reagent (Invitrogen). And then, the cDNA was synthesized from 200 ng extracted total RNA using the SuperScript III^®^ (Invitrogen) and amplified by RT-qPCR based on the TaqMan method on an ABI PRISM 7500 Sequence Detection System (Life Technologies, Grand Island, NY, USA), with the housekeeping gene GAPDH or U6 as an internal control. The 2^–ΔΔCt^ method was used to determine the relative quantification of gene expression levels. All the premier sequences were synthesized by RiboBio (Guangzhou, China), and their sequences are shown as follows: circRNA-0008717 (Forward) 5′-CTAAGGAGTCACAGGAAGACATC-3′, (Reverse) 5′-GTAGAATCTCTCAGACTCAAGGTTG-3′; Bmi-1 (Forward) 5′-AATGTGTGTCCTGTGTGGAGGGT-3′, (Reverse) 5’-GCTGACGGGTGAGCTGCATAAA-3′; GAPDH (Forward) 5′-GAAGGTGAAGGTCGGAGTC-3′, (Reverse) 5′-GAAGATGGTGATGGGATTTC-3′.

### Dual-luciferase reporter assay

The circRNA-0008717 sequence in MG-63 cells was subcloned into the luciferase reporter psiCHECK2 (Promega, Madison, WI) and designated as psiCHECK2-circRNA-0008717-WT. The circRNA-0008717 sequence with mutation of miR-203 binding site was synthesized using overlap extension PCR and cloned into psiCHECK2 vector designated as psiCHECK2-circRNA-0008717-Mut. The Bmi-1 3′ UTR cDNA in MG-63 cells was amplified and cloned to psiCHECK2 and termed as psiCHECK2-Bmi-1–3′ UTR-WT. The mutant vector for miR-203 binding site was constructed and termed as psiCHECK2-Bmi-1–3′ UTR-Mut.

3 × 10^4^ osteosarcoma cells were seeded in 24-well plates in triplicate. Forty-eight h after cotransfection with corresponding plasmids and microRNA mimics, luciferase reporter assays were conducted using Dual-luciferase reporter assay system (Promega, Madison, WI) according to the manufacturer's instructions. Relative luciferase activity was normalized to the Renilla luciferase internal control.

### MTT assay

Cell proliferation was evaluated by using 3-(4,5-Dimethylthiazol-2-yl)-2,5-diphenylt etrazolium bromide (MTT, Sigma, MO, USA). Briefly, 5 × 10^3^ cells per well were seeded into a 96-well plate. After transfection, the optical density was measured at 450 nm using a microtiter plate reader at different time points, and the rate of cell survival was expressed as the absorbance.

### FACS apoptosis assay

Cells (1 × 10^5^/well) were collected 48 h after transfection, and were stained with Annexin V FITC and propidium iodide (PI) according to the manufacturer's instructions (BD Biosciences, Erembodegem, Belgium). Apoptosis was assessed by flow cytometry (BD FACSCalibur).

### Colony formation assays

MG-63 and SAOS-2 cells were infected with sh-circRNA-0008717 and cultured for 72 hours, and then they were re-plated in 6-well plates at the density of 5 × 10^2^ per well and maintained for 10 days. The colonies were fixed and stained with 0.5% crystal violet for 15 minutes.

### Cell migration and invasion assay

Cell migratory ability was detected by wound-healing assay. Briefly, transfected/treated cells were seeded in 6-well plates and cultured to near confluence. Artificial wounds were created on the cell monolayer using cultureinserts for live cells analysis, and then migrated cells and wound healing were visualized. For each group, at least 3 artificial wounds were photographed immediately and at the time points indicated after the wound formation. After the wounds were created, the cells were incubated in culture medium without FBS and then photographed at 48 h. Percent of wound closure was calculated with Image J 1.47 software.

Cell invasive ability was detected by using Transwell permeable supports (Corning, USA) according to manufacturer's protocol. Briefly, the transfected/treated cells were plated onto a Matrigel-coated membrane in the upper chamber of a 24-well insert containing medium free of serum. The bottom chamber contained DMEM with 10% FBS. Cells were incubated at 37°C with 5% CO_2_ for 48 h after plating. Then, the bottom of the chamber insert was fixed with methanol and stained with DAPI for 5 minutes. The number of cells that invaded through the membrane was determined from digital images captured on an inverted microscope and calculated with Image J 1.47 software.

### circRNAs immunoprecipitation (circRIP)

Biotin-labeled circRNA-0008717 probe (5′-CCGTC CGGAACTATGAACAACAATGGCA-3′-biotin) was synthesized by Sangon Biotech and the circRIP assay was performed as previously described with minor modification [44]. MG-63 cells were fixed by 1% formaldehyde for 10 minutes, lysed and sonicated. After centrifugation, 50 μl of the supernatant was retained as input and the remaining part was incubated with circRNA-0008717 specific probes-streptavidin dynabeads (M-280, Invitrogen) mixture over night at 30°C. Next day, M-280 dynabeads-probes-circRNAs mixture was washed and incubated with 200 μl lysis buffer and proteinase K to reverse the formaldehyde crosslinking. Finally, the mixture was added with TRIzol for RNA extraction and RT-qPCR detection.

The RIP experiment using Bmi-1 antibody (1:1000; Santa Cruz Biotechnology, Santa Cruz, CA, USA) to pull down miR-203 was also performed. The Magna RIP RNA-Binding Protein Immunoprecipitation Kit (Millipore, Bedford, MA, USA) was used according to the manufacturer's instructions. Total RNA and controls were also assayed to demonstrate that the detected signals were from RNAs specifically binding to Bmi-1. The final analysis was performed using RT-qPCR and shown as the fold enrichment of miR-203.

### Western blot and antibodies

Osteosarcoma cells were lysed with radioimmunoprecipitation assay (RIPA) buffer (Sigma, MO, USA) containing protease inhibitors (Sigma). Protein quantification was done using a BCA protein assay kit (Promega, USA). The primary antibodies used for western blotting were rabbit anti-human Bmi-1 antibody (sc-390443, 1:500, Santa Cruz Biotechnology) and rabbit anti-human β-actin antibody (#4967, 1:1000, Cell Signaling Technology). Horseradish peroxidase-conjugated (HRP) anti-rabbit antibodies (1:5000; Santa Cruz Biotechnology, Beverly, MA, USA) were used as the secondary antibodies. A total of 25 μg protein from each sample was separated on 10% Bis-Tris polyacrylamide gel through electrophoresis and then blotted onto polyvinylidene fluoride (PVDF) membranes (GE Healthcare, Piscataway, NJ, USA). Then, the membrane was blocked with 5% (5 g/100 mL) nonfat dry milk (Bio-Rad, CA, USA) in tri-buffered saline plus Tween (TBS-T) buffer for 2 h. Blots were immunostained with primary antibody at 4°C overnight and with secondary antibody at room temperature for 1 h. Immunoblots were visualized using Immobilon^TM^ Western Chemiluminescent HRP Substrate (Millipore). Protein levels were normalized to β-actin.

### Statistical analysis

Kolmogorov-Smirnov test was used to determine the normality of the distribution of data in each group. For osteosarcoma vs. normal cell lines, and osteosarcoma tissue vs. adjacent non-tumor tissue, differences were shown in median expression and were determined using the Mann-Whitney *U* test or Kruskal-Wallis test. The correlation analysis was evaluated by using the Spearman test. Receiver operator characteristic (ROC) curve analysis and area and the curve (AUC) was used to determine the diagnostic value of circRNA-0008717. Count dates were described as frequency and examined using Fisher's exact test. A log-rank test was used to analyze the statistical differences in survival as deduced from Kaplan-Meier curves. The results were considered statistically significant at *P* < 0.05. Error bars in figures represent SD (Standard Deviation). Statistical analyses were performed with GraphPad Prism (version 5.01, La Jolla, CA, USA) software. ^*^P < 0.05; ^**^P < 0.01; ^***^P < 0.001.
